# Paternal and Maternal History of Myocardial Infarction and Cardiovascular Diseases Incidence in a Dutch Cohort of Middle-Aged Persons

**DOI:** 10.1371/journal.pone.0028697

**Published:** 2011-12-16

**Authors:** Ineke van Dis, Daan Kromhout, Jolanda M. A. Boer, Johanna M. Geleijnse, W. M. Monique Verschuren

**Affiliations:** 1 Netherlands Heart Foundation, The Hague, The Netherlands; 2 Division of Human Nutrition, Wageningen University, Wageningen, The Netherlands; 3 National Institute for Public Health and the Environment (RIVM), Bilthoven, The Netherlands; University Institute of Social and Preventive Medicine, Switzerland

## Abstract

**Background:**

A positive parental history of myocardial infarction (MI) is an independent risk factor for cardiovascular diseases (CVD). However, different definitions of parental history have been used. We evaluated the impact of parental gender and age of onset of MI on CVD incidence.

**Methods:**

Baseline data were collected between 1993 and 1997 in 10 524 respondents aged 40–65 years. CVD events were obtained from the National Hospital Discharge Register and Statistics Netherlands. We used proportional hazard models to calculate hazard ratios (HR) and 95% confidence intervals (CI) for CVD incidence and adjusted for lifestyle and biological risk factors.

**Results:**

At baseline, 36% had a parental history of MI. During 10-year follow-up, 914 CVD events occurred. The age and gender adjusted HR was 1.3 (95% CI 1.1–1.5) for those with a paternal MI, 1.5 (1.2–1.8) for those with a maternal MI and 1.6 (1.2–2.2) for those with both parents with an MI. With decreasing parental age of MI, HR increased from 1.2 (1.0–1.6) for age ≥70 years to 1.5 (1.2–1.8) for age <60 years for a paternal MI and from 1.1 (0.9–1.5) to 2.2 (1.6–3.0) for a maternal MI. The impact of having a mother with MI before age 60 significantly differed in women [(2.9 (1.8–4.6)] and men [1.5 (0.9–2.6)]. Adjustment only slightly influenced HRs for maternal MI.

**Conclusions:**

Respondents with a parental history of MI have an increased CVD incidence, in particular with parental onset of MI before age 70. A maternal history of MI before age 60 was the strongest predictor of CVD incidence.

## Introduction

A positive parental history of a myocardial infarction (MI) is an independent risk factor for cardiovascular diseases (CVD). This relationship was already demonstrated in the 1970s in men [1] and in the 1980s in women [2], and has been confirmed in many prospective studies. However, most studies were conducted in men only and parental history was not uniformly defined. Simple definitions were used as having a parent, a father or a mother, or both parents with a history of MI, regardless of the age of onset. Relative risks of 1.0–2.2 in men and women were found [3], [4], [5], [6], [7], [8], [9], [10], [11], [12], [13]. Some studies showed a slightly stronger paternal [4], [12], [13] and others a stronger maternal transmission [3], [5], [6], [9] in men. In women, only a few studies were conducted [6], [8], [9]. For both parents with a history of MI, regardless of the age of onset, a relative risk of approximately 2 was observed [6].

In most studies on the risk of premature parental MI, the cut-off for the age of onset of the MI was fixed and ranged from a parental MI before the age of 65 to before age 50 [1], [5], [9], [14], [15], [16], [17]. The National Cholesterol Education Program Third Adult Treatment Panel (NCEP ATP III) recommended that cut-off points should be age of onset before age 65 in the father and before age 55 in the mother [18]. These different cut points for men and women were applied in several studies [19], [20], [21]. Relative risks of premature parental history of MI amounted to 1.2–2.8 in men and women. As for parental history irrespective of age of onset, differences between paternal and maternal transmission were inconsistent, and for those with both parents who had had a premature MI, relative risks of 1.5–4.1 were found [4], [16], [21], [22].

The age of onset of MI was studied in more detail in two large US cohorts [6], [22]. These studies showed a continuous increase in relative risks of CVD with decreasing paternal or maternal age of onset of the MI. In men, a paternal and maternal MI before age 70 [6], [22] conferred a greater risk of CVD (RR 1.7–2.6) compared to those with a higher parental age of MI. For women this was the case for a paternal and maternal MI below age 50 [6].

An important question is, whether the positive relationship between parental history of MI can be explained by lifestyle factors such as smoking, alcohol intake and physical inactivity and/or risk factors such as blood pressure and serum cholesterol. In most studies, adjusting for lifestyle [4], [5], [6] and risk factors [14], [15], [16], [19], [20], [21] only slightly lowered the relative risk in respondents with (premature) parental MI.

The aim of the present study was to investigate the relationship between various definitions of parental history of MI and CVD incidence in the offspring, with emphasis on the impact of a maternal and paternal MI and on older or younger parental age of onset of the MI. Furthermore we investigated the impact of lifestyle and risk factors on the association between parental history and CVD incidence.

## Methods

### Study population

In the Monitoring Project on Chronic Disease Risk Factors (MORGEN-project), baseline cardiovascular risk factor data were collected in 1993–1997 in 22 769 men and women aged 20–65 years [23], [24]. The project was carried out in random population samples of Amsterdam (respons rate 33%), Maastricht (43%) and Doetinchem (68%) in the Netherlands. We used only data of respondents aged 40–65 years (12 328), because many younger respondents will have parents who were too young to have experienced an MI. The survey was approved by the Medical Ethics Committee of TNO Prevention and Health, Leiden and respondents signed an informed consent form.

### Data collection

Information on parental history of MI, demographic variables, lifestyle factors, educational level and disease history was obtained by standardized questionnaires. Parental history of MI was ascertained using the questions ‘did your father ever experience an MI’ and ‘did your mother ever experience an MI’, followed by a question about the age of onset for those reporting a parental MI. We categorized respondents into four categories of parental history: both parents without MI (reference group), a father only with MI, a mother only with MI or both parents with MI. In order to study the effect of age of onset of MI, and to define premature MI, the age of onset of the parental MI was classified as ≥70 years, 60–69 years and <60 years for both the mother and the father. This classification allowed for a proper number of respondents in each parental age of onset of MI category, especially in those having a mother with MI. In a reproducibility study in part of our respondents, 85% of respondents were classified into the same parental history group again (no, one or two parents with MI<60 years in men and <65 years in women). Smoking was dichotomized as ‘current smoker’ or ‘non-smokers’ (including ex-smokers). Alcohol consumption was defined as none, moderate (male: ≤2 glasses/female ≤1 glass per day) and high (male: >2 glasses/female >1 glass per day). Physical activity was assessed by a validated questionnaire in respondents recruited between 1994 and 1997. For this subset, we calculated whether respondents practised cycling and sports with a metabolic equivalent score ≥4 (yes/no) [25]. Educational level was classified into three categories: low (intermediate secondary education or less), intermediate (intermediate vocational or higher secondary education) or high (higher vocational or university education). The prevalence of MI or diabetes mellitus at baseline (yes/no) was based on self-report.

Body weight and height were measured by trained staff according to the WHO recommendations. BMI was calculated by dividing weight (in kg) by height (in m) squared [26]. Non-fasting blood samples were taken and serum total cholesterol was measured by the CHOD-PAP method. HDL-cholesterol was determined in the supernatant after precipitation of apo B-containing lipoproteins with magnesium phosphotungstate [27]. All cholesterol determinations were done in a standardized laboratory [23]. Blood pressure was measured twice by a trained technician at the right upper arm with the participant in sitting position using a random zero sphygmomanometer. Systolic blood pressure was recorded at the first, and diastolic blood pressure at the fifth Korotkoff phase. The mean of both measurements was used in the analyses.

### Mortality and morbidity follow-up

The cohort was linked to three registries. Vital status was checked by the national population register. Only 18 persons were lost to follow-up and 538 persons migrated. Data on CVD mortality during follow-up were obtained from Statistics Netherlands (till 1 January 2006). CVD morbidity was identified through linkage with the National Hospital Discharge Register (till 1 January 2006) based on information on the date of birth, gender and postal code using a validated probabilistic method [24]. Record linkage was in 98% of the respondents successful [24]. Total CVD was defined as the first event during 10-year follow-up, either non-fatal or fatal, whichever came first. Cases were censored at the first date of hospital admission for CVD or at the date of death. Other subjects were censored at the date of emigration, date of death, at the end of follow-up or at a maximum of 11 years, resulting in a mean follow-up of 10 years. We defined fatal and non-fatal events identical to the ICD-codes used in the SCORE-project: CVD (ICD 9: 401–414, 426–443, 798.1, 789.2, ICD 10: I10–I25, I46, I47–I51, I61–I65, G45, I67–I69, I70–I72, R96) [28]. In total, 914 events occurred, of which 40 were fatal without a previous non-fatal event and 47 were fatal preceded by a non-fatal event. Most events were due to coronary heart diseases (557), peripheral arterial diseases (128), stroke (125), heart failure (60) and hypertensive ischemic heart diseases (40).

### Data and statistical analyses

We excluded respondents without informed consent for linkage with registries, respondents under the age of 40, prevalent cases of MI and respondents with missing data on parental history of MI and age of onset, on lifestyle and risk factors. This left 10 524 respondents, 4878 men and 5646 women for analysis. First we investigated whether there was interaction between gender and parental history of MI. Since there was no significant interaction (p = 0.22) for most definitions of parental history of MI, we pooled the data of men and women and adjusted for gender. There was interaction with gender only for those respondents whose mother had an MI before age 60 (p = 0.04).

Baseline characteristics of the study population by parental MI - subdivided by paternal, maternal or both parents with MI - were described as mean and standard deviation for normally distributed continuous variables and numbers and percentages for categorical variables. The 10-year cumulative incidence rates according to parental history of MI were calculated.

Associations between parental history of MI and CVD incidence were analyzed with Cox proportional hazard models. The proportionality of the hazards was verified by plotting the log-log survival curves and graphical inspection of parallelism of the curves. In all analyses respondents with ‘both parents without an MI’ were used as the reference category. All hazard ratios (HR) were adjusted for age and gender. The HR's for maternal MI were stratified by gender, adjusted for paternal MI and included an interaction term mother*gender in the model. We additionally adjusted for education and the following lifestyle-related factors: current smoking status, alcohol intake and BMI. In the main analyses we did not adjust for physical activity because this variable was not estimated in respondents recruited in 1993 and adjustment for physical activity in the respondents recruited from 1994–1997 only slightly attenuated the HRs. Furthermore, we adjusted for the risk factors serum total and HDL-cholesterol, systolic blood pressure (continuously) and for self-reported diabetes mellitus (dichotomously).

For all analyses SAS 9.2 (SAS Institute Inc., Cary, North Carolina, USA) 2002–2008 was used.

## Results

At baseline, 36% of the men and women had one or two parents who experienced an MI (23% only a father with MI, 8% only a mother with MI and 4% both parents) (table 1). Age of respondent was not associated with parental history of MI, while serum total cholesterol and the prevalence of self-reported diabetes were highest in those with two parents with an MI. Average systolic blood pressure was highest in men with a maternal history of MI. The number of current smokers was lowest in men with two parents with an MI. A high level of education was more common in respondents with parents without MI and those with a father with MI. Fathers experienced their MI at a mean age of 63, mothers at age 67.

**Table 1:** Baseline characteristics* and observed percentage of CVD** in 4878 men and 5646 women by parental MI in the MORGEN-cohort.

	Men				Women			
Parents MI:	No parents	Father only	Mother only	Both parents	No parents	Father only	Mother only	Both parents
Number	3149	1210	342	177	3618	1260	511	257
Age (years,sd)	50.6 (6.2)	49.6 (6.0)	50.6 (5.9)	50.7 (5.8)	50.3 (6.3)	49.8 (6.0)	51.2 (6.3)	51.0 (5.8)
High education, %	29	29	23	20	19	18	12	15
Smokers, %	33.0	31.8	34.5	27.7	31.9	36.8	35.6	37.0
Moderate alcohol intake, %	53.5	54.8	51.8	53.7	54.1	52.5	56.2	51.4
Cycling, %	74.9	72.2	71.2	76.7	77.6	75.6	75/7	71.5
Sports, %	38.1	37.4	35.6	28.6	36.9	38.8	31.5	33.2
BMI (kg/m^2^, sd)	26.1 (3.5)	26.2 (3.5)	26.3 (3.4)	26.3 (3.3)	25.5 (4.3)	25.4 (4.0)	25.8 (4.5)	25.6 (4.4)
Systolic BP (mmHg, sd)	127.3 (16.3)	127.5 (15.2)	129.4 (16.5)	128.9 (15.4)	121.7 (17.5)	122.2 (17.2)	123.5 (17.8)	124.4 (17.0)
Serum total cholesterol (mmol/l, sd)	5.57 (0.99)	5.67 (1.03)	5.65 (0.99)	5.77 (0.92)	5.55 (1.04)	5.59 (1.04)	5.72 (1.00)	5.78 (1.05)
Serum HDL-cholesterol (mmol/l, sd)	1.21 (0.31)	1.17 (0.31)	1.16 (0.31)	1.15 (0.27)	1.53 (0.39)	1.55 (0.40)	1.49 (0.37)	1.46 (0.36)
Self reported diabetes, %	1.6	1.2	1.8	2.3	1.3	1.1	1.6	2.0
Paternal age of MI (years)	-	62.3	-	63.9	-	62.9	-	64.3
Maternal age of MI (years)	-	-	68.2	67.4	-	-	66.3	66.9
CVD, n, %	353 (11.2)	173 (14.3)	48 (14.0)	26 (14.7)	177 (4.9)	68 (5.4)	43 (8.4)	26 (10.1))

*Data are presented as mean, SD or %.

**CVD events during an average follow-up of 10 years.

BMI, body mass index; HDL, high density lipoproteïn; MI, myocardial infarction; CVD, cardiovascular diseases.

**Figure 1:**
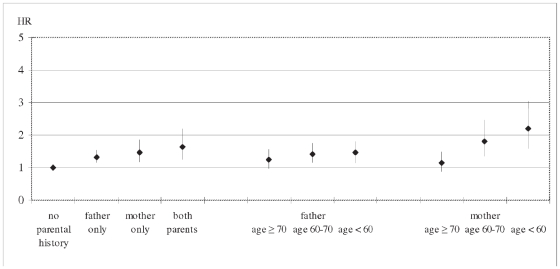
Hazard ratio* of CVD and 95% confidence intervals by parental age of onset of MI in respondents aged 40–65 years. * HR adjusted for age and gender.

All three groups with a positive parental history of MI had an increased CVD risk compared to those without a parental history of MI (table 2, fig. 1). The age and gender adjusted pooled HR was 1.3 (95% CI 1.1–1.5) for having only a father, 1.5 (1.2–1.8) for having only a mother and 1.6 (1.2–2.2) for having both parents with MI. With decreasing parental age of MI, the HR for a paternal MI increased from 1.2 (1.0–1.6) for age ≥70 years to 1.5 (1.2–1.8) for age <60 years and for a maternal MI from 1.1 (0.9–1.5) to 2.2 (1.6–3.0).

**Table 2:** Adjusted hazard ratios of CVD (HR (95% CI) in respondents aged 40–65 years by parental gender and parental age at onset of MI.

	No parental history	FatherMI only	Mother MI only	Both parentsMI	FatherMI≥70 y	FatherMI 60–69 y	FatherMI<60 y	Mother MI≥70 y	Mother MI 60–69 y	Mother MI<60 y
n	6767	2470	853	434	852	962	1090	612	400	275
Model 1	1	1.33(1.14–1.55)	1.47(1.18–1.84)	1.64(1.24–2.18)	1.24(0.98–1.56)	1.41(1.14–1.74)	1.47(1.20–1.81)	1.14(0.87–1.50)	1.82(1.34–2.46)	2.20(1.59–3.04)
Model 2	1	1.33(1.14–1.55)	1.41(1.13–1.76)	1.66(1.25–2.21)	1.27(1.01–1.60)	1.43(1.16–1.77)	1.42(1.16–1.75)	1.16(0.88–1.52)	1.65(1.21–2.23)	2.10(1.52–2.90)
Model 3	1	1.30(1.12–1.52)	1.33(1.06–1.66)	1.55(1.16–2.06)	1.27(1.01–1.60)	1.38(1.11–1.71)	1.37(1.11–1.68)	1.09(0.83–1.43)	1.52(1.12–2.07)	2.05(1.48–2.84)

Model 1: adjusted for age and gender.

Model 2: model 1+adjusted for smoking, alcohol intake, BMI and education.

Model 3: model 2+adjusted for systolic blood pressure, serum total and HDL-cholesterol, self reported diabetes mellitus.

**Figure 2:**
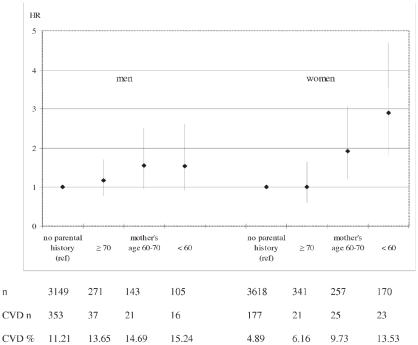
Hazard ratio* of CVD and 95% confidence intervals by mother's age of onset of MI in men and women aged 40–65 years. * HR adjusted for age and father's age of onset of MI.

No interaction between gender and parental history of MI was found, except an interaction between gender and maternal history of MI at age below 60 (P = 0.04). Compared to women without a parental history of MI, the HR was 1.0 (0.6–1.6) for women having a mother with age of onset of MI above 70 years, 1.9 (1.2–3.1) at age 60–69 years and 2.9 (1.8–4.6) at age below 60 (fig. 2). Compared to men without a parental history of MI, these HRs were 1.2 (0.8–1.7), 1.6 (1.0–2.5) and 1.5 (0.9–2.6) respectively in men.

In multivariable analyses - adjusting for age, gender, education and lifestyle-related factors such as smoking, physical activity (in subset), alcohol intake and BMI - HRs for paternal MI were of the same magnitude compared to those adjusted only for age and gender, and slightly lower for maternal MI. Additional adjustment for biological risk factors yielded similar HRs for paternal MI and slightly attenuated the HRs for maternal MI at age of onset below 60 years (table 2).

## Discussion

Respondents with a parental history of MI had a 30–60% higher CVD risk, after adjustment for age and gender, regardless of the age at which their parents had an MI. Parental history of MI was considered premature at onset of the MI before age 70, based on significantly increased risks. In cases of premature parental MI, a maternal transmission of CVD risk was twice as strong (80–120% increased risk) as paternal transmission (40–50%). The impact of a maternal MI at age below 60 was twice as strong as in women (HR 2.9) than in men (HR 1.5). These associations could not be explained by higher levels of lifestyle and biological risk factors in respondents with a paternal history of MI and only to a small extent in those with a maternal history of MI.

The strength of the present study was that we had detailed information on parental history of MI for men and women. The vast majority of studies on the relationship of parental history of MI with CVD incidence reported on men only. We did not find an interaction between gender and parental history of MI, so our data suggest that results did not differ between men and women. We had also information available on the age of onset of MI in both fathers and mothers. Only a few studies investigated parental MI according to the age of onset of parental MI and CVD incidence beyond cut-off points for premature history of MI [6], [22]. We reported on respondents aged 40–65 years, because younger respondents had parents who were too young to have experienced a MI. However, even in the selected age group of respondents, some parents were too young to have experienced a MI, especially mothers. Mean age of MI in mothers was four years lower than in fathers.

Information on non-fatal CVD was obtained from hospital discharge data through record linkage with the National Hospital Discharge Register. The linkage success rate for cardiovascular endpoints was 97.6% [24]. Comparison of hospital discharge diagnoses for a sub sample from the Maastricht area with a cardiology information system in this region, showed that 14.2% of the cases of acute myocardial infarction were not registered in the hospital discharge register [29]. The reliability of the cause-of-death coding was examined by Statistics Netherlands. The inter-coder agreement of the underlying cause of death by ICD-10 code was 89.1% for acute myocardial infarction and 78.5% for stroke [30]. This minor underestimation and misclassification of non-fatal and fatal endpoints probably did not affect the association between parental history of MI and CVD incidence.

We studied the impact of parental history of MI on nonfatal and fatal CVD incidence. This broad endpoint definition was the same as the one used in the SCORE-project and included myocardial infarction (21%), angina pectoris (20%), cerebrovascular accidents (14%), peripheral arterial diseases (14%), congestive heart failure (7%) and hypertensive heart diseases (4%). Similar broad definitions of CVD were also used in some studies [6], [14], [21], while almost all studies included non-fatal and fatal CHD [5], [9], [12], [13], [16], [22]. However, similar relative risks were observed for parental history of MI in relation to different definitions of CVD endpoints, suggesting similar impacts on CHD and CVD incidence.

As in most studies, in the present study parental history of MI was based on self-report by the respondents. In 1995, a reproducibility study was carried out. Data on paternal and maternal history of myocardial infarction were obtained by questionnaire from 241 participants from Maastricht (out of 480 eligible, response 50.2%). The general agreement of paternal and maternal history of MI was 88.8% and 85.9%, respectively, while the κ-statistic was 0.80 for paternal and 0.73 for maternal history of MI. The discrepancy in reported age was two years or less for 69% of the paternal events and 65% of the maternal events, while the discrepancy was more than 5 years for 8% of the paternal and 13% of the maternal events.

Several validation studies were carried out by others, in which parental events were confirmed by medical records [31], [32], [33]. Parental history of MI or CHD was correctly reported by 68%–89% of respondents. In the Framingham Heart Study, in which both off-spring and parental CVD events were validated, higher relative risks for a parental history of MI were found [21]. This suggests that stronger relative risks are obtained when more accurate information on parental history of MI is used.

Our results confirm previous findings of an independent relationship between parental history of MI and CVD incidence. For the ‘simple’ definitions of having a father, a mother or both parents with a history of MI, we found HRs of 1.3, 1.5 and 1.6 respectively, which were in the same order of magnitude as observed in other studies (RR 1.2–2.2) [3], [4], [5], [6], [7], [8], [9], [10], [11], [12], [13]. Furthermore, we showed a strong gradient of CVD incidence with decreasing parental age of onset of MI. CVD incidence was significantly increased when a father or a mother experienced an MI under the age of 70. This cut-off point of a premature parental history of MI was comparable to that found in US Physicians' Health and Health Professionals studies [6], [22]. However, the cut-off points for increased CHD risk set by the NCEP expert panel (fathers who were younger than 65 at the age of onset and mothers who were under 55 at the age of onset) [18] and the fixed cut-off points used in most other studies for premature parental MI (at or before the age of 65) [1], [9], [14], [15], [16], [21] are at younger age compared to the present study, especially those for women. We observed a stronger maternal than paternal transmission of risk for different ages of onset of an MI. However, other studies on (premature) parental MI found either a stronger paternal transmission [4], [7], [12], [15], [16], stronger maternal transmission [3], [5], [6], [9] or no difference [2], [21], [22] in men. So definitive conclusions cannot be drawn on this issue.

In the present study, adjusting for education, lifestyle and risk factors did not influence the CVD incidence for paternal MI, and only slightly attenuated the CVD incidence associated with maternal MI. Similar results were found in other studies on lifestyle [4], [5], [6] and risk factors [5], [14], [15], [16], [17], [19], [20], [21] This means that the relationship between a positive family history and CVD incidence is largely independent from known lifestyle and risk factors. Therefore, this relationship may be due to genetic factors. However, influence of unknown or unmeasured lifestyle and biological factors cannot be excluded.

In conclusion, individuals with a parental history of MI had an increased CVD incidence of similar magnitude in men and women. CVD risk increased gradually with decreasing parental age of onset of MI. Based on our results, a premature parental MI can be defined as having a father and/or a mother with MI before the age of 70. Higher age cut-off points for defining premature parental MI might be warranted. The strongest associations were observed in persons with a mother who had an MI before age 60, especially in women.
